# The Effects of Influenza Vaccination of Health Care Workers in Nursing Homes: Insights from a Mathematical Model

**DOI:** 10.1371/journal.pmed.0050200

**Published:** 2008-10-28

**Authors:** Carline van den Dool, Marc J. M Bonten, Eelko Hak, Janneke C. M Heijne, Jacco Wallinga

**Affiliations:** 1 Julius Center for Health Sciences and Primary Care, University Medical Center Utrecht, Utrecht, The Netherlands; 2 Department of Medical Microbiology, University Medical Center Utrecht, Utrecht, The Netherlands; 3 Center for Infectious Disease Control, National Institute for Public Health and the Environment, Bilthoven, The Netherlands

## Abstract

**Background:**

Annual influenza vaccination of institutional health care workers (HCWs) is advised in most Western countries, but adherence to this recommendation is generally low. Although protective effects of this intervention for nursing home patients have been demonstrated in some clinical trials, the exact relationship between increased vaccine uptake among HCWs and protection of patients remains unknown owing to variations between study designs, settings, intensity of influenza seasons, and failure to control all effect modifiers. Therefore, we use a mathematical model to estimate the effects of HCW vaccination in different scenarios and to identify a herd immunity threshold in a nursing home department.

**Methods and Findings:**

We use a stochastic individual-based model with discrete time intervals to simulate influenza virus transmission in a 30-bed long-term care nursing home department. We simulate different levels of HCW vaccine uptake and study the effect on influenza virus attack rates among patients for different institutional and seasonal scenarios. Our model reveals a robust linear relationship between the number of HCWs vaccinated and the expected number of influenza virus infections among patients. In a realistic scenario, approximately 60% of influenza virus infections among patients can be prevented when the HCW vaccination rate increases from 0 to 1. A threshold for herd immunity is not detected. Due to stochastic variations, the differences in patient attack rates between departments are high and large outbreaks can occur for every level of HCW vaccine uptake.

**Conclusions:**

The absence of herd immunity in nursing homes implies that vaccination of every additional HCW protects an additional fraction of patients. Because of large stochastic variations, results of small-sized clinical trials on the effects of HCW vaccination should be interpreted with great care. Moreover, the large variations in attack rates should be taken into account when designing future studies.

## Abstract 2

Using a mathematical model to simulate influenza transmission in nursing homes, Carline van den Dool and colleagues find that each additional staff member vaccinated further reduces the risk to patients.

## Editors' Summary

**Background.:**

Every winter, millions of people catch influenza, a contagious viral disease of the nose, throat, and airways. Most people recover completely from influenza within a week or two but some develop life-threatening complications such as bacterial pneumonia. As a result, influenza outbreaks kill about half a million people—mainly infants, elderly people, and chronically ill individuals—each year. To minimize influenza-related deaths, the World Health Organization recommends that vulnerable people be vaccinated against influenza every autumn. Annual vaccination is necessary because flu viruses continually make small changes to the viral proteins (antigens) that the immune system recognizes. This means that an immune response produced one year provides only partial protection against influenza the next year. To provide maximum protection against influenza, each year's vaccine contains disabled versions of the major circulating strains of influenza viruses.

**Why Was This Study Done?:**

Most Western countries also recommend annual flu vaccination for health care workers (HCWs) in hospitals and other institutions to reduce the transmission of influenza to vulnerable patients. However, many HCWs don't get a regular flu shot, so should efforts be made to increase their rate of vaccine uptake? To answer this question, public-health experts need to know more about the relationship between vaccine uptake among HCWs and patient protection. In particular, they need to know whether a high rate of vaccine uptake by HCWs will provide “herd immunity.” Herd immunity occurs because, when a sufficient fraction of a population is immune to a disease that passes from person to person, infected people rarely come into contact with susceptible people, which means that both vaccinated and unvaccinated people are protected from the disease. In this study, the researchers develop a mathematical model to investigate the relationship between vaccine uptake among HCWs and patient protection in a nursing home department.

**What Did the Researchers Do and Find?:**

To predict influenza virus attack rates (the number of patient infections divided by the number of patients in a nursing home department during an influenza season) at different levels of HCW vaccine uptake, the researchers develop a stochastic transmission model to simulate epidemics on a computer. This model predicts that as the HCW vaccination rate increases from 0 (no HCWs vaccinated) to 1 (all the HCWs vaccinated), the expected average influenza virus attack rate decreases at a constant rate. In the researchers' baseline scenario—a nursing home department with 30 beds where patients come into contact with other patients, HCWs, and visitors—the model predicts that about 60% of the patients who would have been infected if no HCWs had been vaccinated are protected when all the HCWs are vaccinated, and that seven HCWs would have to be vaccinated to protect one patient. This last figure does not change with increasing vaccine uptake, which indicates that there is no level of HCW vaccination that completely stops the spread of influenza among the patients; that is, there is no herd immunity. Finally, the researchers show that large influenza outbreaks can happen by chance at every level of HCW vaccine uptake.

**What Do These Findings Mean?:**

As with all mathematical models, the accuracy of these predictions may depend on the specific assumptions built into the model. Therefore the researchers verified that their findings hold for a wide range of plausible assumptions. These findings have two important practical implications. First, the direct relationship between HCW vaccination and patient protection and the lack of any herd immunity suggest that any increase in HCW vaccine uptake will be beneficial to patients in nursing homes. That is, increasing the HCW vaccination rate from 80% to 90% is likely to be as important as increasing it from 10% to 20%. Second, even 100% HCW vaccination cannot guarantee that influenza outbreaks will not occasionally occur in nursing homes. Because of the large variation in attack rates, the results of small clinical trials on the effects of HCW vaccination may be inaccurate and future studies will need to be very large if they are to provide reliable estimates of the amount of protection that HCW vaccination provides to vulnerable patients.

**Additional Information.:**

Please access these Web sites via the online version of this summary at http://dx.doi.org/10.1371/journal.pmed.0050200.

- Read the related *PLoSMedicine* Perspective by Cécile Viboud and Mark Miller
- A related *PLoSMedicine* Research Article by Jeffrey Kwong and colleagues is also available
- The World Health Organization provides information on influenza and on influenza vaccines (in several languages)
- The US Centers for Disease Control and Prevention provide information for patients and professionals on all aspects of influenza (in English and Spanish)
- The UK Health Protection Agency also provides information on influenza
- MedlinePlus provides a list of links to other information about influenza (in English and Spanish)
- The UK National Health Service provides information about herd immunity, including a simple explanatory animation
- The European Centre for Disease Prevention and Control provides an overview on the types of influenza

## Introduction

Annual influenza vaccination of institutional health care workers (HCWs) is advised in most Western countries to reduce transmission of influenza to vulnerable patients [1]. A few clinical trials have indeed demonstrated protective effects of this intervention for patients in nursing homes at relatively low HCW vaccine uptake rates [2–5]. However adherence to the recommendation is generally low [6–9] and it is uncertain what the effect of a further increase of vaccine uptake among HCWs is and whether herd immunity can be attained in health care institutions [10]. Empirical data from previous trials did not reveal a clear association between the number of HCWs vaccinated and the number of prevented influenza virus infections in patients. This absence of a clear association might be due to substantial variation between the studies in the endpoints measured, the departments (of varying size) involved, and vaccine coverage among patients. Furthermore, the effect of HCW vaccination is highly dependent on annual factors such as influenza virus activity and the match between the circulating influenza virus strain and the current vaccine. To control for all uncertainties potentially modifying the effects of HCW vaccination an exceptionally large clinical trial would be needed.

We, therefore, propose to disentangle the impact of effect modifiers with a mathematical model that can simulate the occurrence of influenza virus infections in a nursing home department under various vaccination and institutional scenarios. With the model we aim to elucidate the relationship between HCW vaccination and patient attack rates for a given department size, vaccine uptake among patients, and vaccine efficacy. Furthermore, we explore whether herd immunity can be expected to occur in a nursing home at higher levels of HCW vaccine uptake.

## Methods

### Population and Model

We simulate the occurrence of influenza virus outbreaks in a typical Dutch long-term care nursing home department with 30 beds (in 15 two-bed rooms) and a team of 30 HCWs. We assume the 30 HCWs work in shifts of 8 h according to a weekly schedule, with five HCWs working during the day shift, three during the evening, and one during the night. As we are simulating a small population where chance events can have major effects we use a stochastic transmission model. Below we describe the essential structure of this model; a detailed description is presented in the Text S1.

### Infection Cycle

According to a standard model for infectious disease transmission, individuals can be in one of several stages of influenza virus infection: susceptible, infected but not yet infectious (exposed), infectious, or recovered/immune (S, E, I, or R) [11,12]. Susceptible individuals can be infected and become exposed through contact with infectious individuals from either inside or outside the department. After a latent period the exposed individuals become infectious and can infect others until they recover and become immune. Individuals acquire immunity either by recovery after infection or by vaccination prior to the influenza season, and immune individuals do not return to the susceptible pool for the remaining season.

### Influenza Vaccination

Both patients and HCWs can receive influenza vaccine prior to the influenza season. We assume vaccination leads to perfect immunity against infection in a fraction *ve*_1_ of vaccinated patients and *ve*_2_ of vaccinated HCWs, where *ve*_1_ and *ve*_2_ are the vaccine efficacies in the corresponding populations. In the remaining (1 − *ve*_i_) vaccinated individuals the vaccine has no effect. In Text S1 (Figure IX) we show that an alternative assumption (vaccination reduces the probability of becoming infected for all vaccinated individuals, but does not lead to complete immunity) leads to qualitatively similar results.

### Contacts

As described above, susceptible individuals can become infected through contacts with infectious individuals. Therefore, an individual's risk of being infected depends on the frequency of contacts that are made, and the likelihood that the contacted persons are infectious. The number of potential contacts of patients and HCWs varies per shift (day, evening, night). Patients can have contact with other patients, HCWs, and visitors during day and evening shifts. During night shifts, patients can only contact their roommate and HCWs. HCWs can have contacts with other HCWs working in the same shift and with all patients. We distinguish between casual and close contacts. Individuals have a casual contact when they have a conversation, and a close contact when physical contact is present. We parameterize the contact model such that the expected numbers of contacts, specified by type of individuals and kind of contact, matches the number of contacts that we observed in two nursing home departments in the Netherlands (Text S1 [Tables III–V]).

### Transmission

The occurrence of transmission between contacts is modeled by sampling from a Bernoulli distribution with mean set equal to the transmission probability. For every pair of individuals with a casual or close contact, there is a probability *p*_1_ or *p*_2_, respectively, that the virus is transmitted if the individuals involved in the contact are infectious and susceptible. We take *p*_2_ always larger than *p*_1_ to reflect that transmission is more likely for close contacts than for casual contacts.

### Influenza in the Community

The rate at which influenza virus is introduced into the nursing home by HCWs, visitors, and patients depends on the prevalence of the virus in the community. We simulate an influenza epidemic in a large population (the community) with a deterministic SIR model (see Text S1 [Figure I]), and use the associated daily incidence and prevalence rates in our nursing home model. HCWs are assumed to have many contacts in the community and the hazard rate of becoming infected outside the nursing home is equal to the hazard rate of infection in the community. Visitors and new patients are chosen at random from the community and the probability that they are infected when they enter the nursing home is equal to the community prevalence.

### Parameters and Uncertainty Analyses

In the model we use three different parameter types (Table 1): (1) fixed parameters that have the same value in all simulations; (2) uncertain parameters that we vary in an uncertainty analysis; and (3) control parameters that we vary to study different scenarios.

**Table 1:**
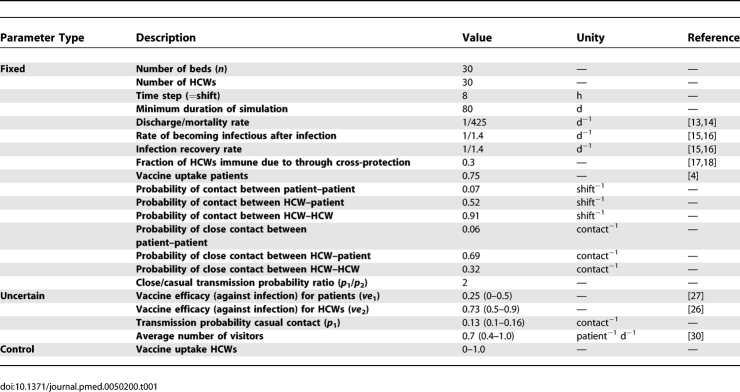
Parameter Values

#### Fixed parameters.

We simulate a period of at least 80 d to cover the length of a typical national influenza epidemic. If there are still infected individuals in the department after 80 d, the simulation is continued until no infected individuals are left. We take time steps of 8 h, equal to the length of a HCW's shift. The patients' average length of stay in the department is 14 mo [13,14]. The durations of the latent and infectious periods are exponentially distributed with means of 1.4 d such that the resulting generation time equals 2.8 d, which is in agreement with observations of generation times during influenza epidemics [15,16]. At the start of the influenza season, 30% of the adult population is assumed to be immune to infection due to cross protection from earlier infections (see also Text S1) [17,18]. The elderly however have a weakened immune system [19,20] and thus we assume absence of immunological memory of previous infections. In correspondence with a recently published HCW vaccination trial, on average 75% of the nursing home patients have been vaccinated [4].

The contact rates between HCWs and patients are based on observations of contact behavior in nursing homes. We determine the probability of contact between two individuals given their type (HCW or patient) as well as the probability that this contact is a close contact (involving physical contact) (Text S1 [Tables III–V]). During their working shift, the probability that a specific HCW contacts a specific patient is 0.52; the probability that such a contact is a close contact is 0.69. The probability that a specific HCW at work contacts another HCW on the same shift is 0.91, and the probability that this contact is close is 0.31. HCWs do not contact visitors in the department. The probability that a specific patient contacts a specific HCW at work is consistent with the contact behavior of HCWs as described above. The probability that a specific patient contacts another specific patient is 0.13 during the day and evening shifts, and these contacts are close with a probability of 0.06. During the night shift, patients contact their room mate, which is assumed to be a casual contact. During the day and evening shifts, patients can also contact visitors. All contacts with visitors are close.

#### Uncertain parameters.

Uncertainty in parameters is handled by Latin hypercube sampling as was first introduced by McKay et al. [21] and subsequently used for disease transmission models by Blower et al. [22–25]. For the parameters patient vaccine efficacy, HCW vaccine efficacy, transmission probability, and visitor frequency, we choose a likely range for the parameter values (see Table 1) and draw actual values from a uniform distribution over this range. For every scenario under study we make 50 different parameter sets such that the whole range of possible values for each of the four parameters is represented equally.

Vaccine efficacy for healthy adults was estimated as 73% (95% confidence interval (CI) 53%–84%), and therefore we use a range of values between 50% and 90% [26]. For elderly nursing home patients the observed vaccine efficacy against influenza virus infection was not significantly different from zero [27]. However, as other evidence shows that the vaccine protects against influenza illness and complications [27,28] we assume patient efficacy to be between 0% and 50%.

Since no data are available on the probability of transmission for a given contact, we varied the transmission probability parameter and determined the resulting infection attack rates. We choose the transmission probability to be between 0.1 and 0.15 for a casual contact (per shift), such that the expected infection attack rate among patients in the absence of HCW vaccination is 23%, corresponding to observed influenza-like-illness attack rates in a moderate influenza season [4,29]. The probability of transmission for close contacts is twice as large as for casual contacts. Varying this value from 1.5 to 2.5 gives qualitatively similar results, see Text S1 (Figure X). The expected number of visitors was estimated from a Dutch study on nursing home patients and visitors to be between 0.4 and 1.0 patient^−1^ day^−1^ [30].

To assess the variation in outcome due to stochasticity in the transmission process, we perform simulations with a single, default, parameter set (vaccine efficacy HCWs 73%, vaccine efficacy patients 25%, transmission probability 0.13, and the expected number of visitors 0.7, see Text S1) and compare the resulting variance with the one from the baseline simulation that uses the whole described parameter space.

#### Control parameters.

Simulations are performed for rates of HCW vaccine uptake of 0, 0.25, 0.5, 0.75, and 1. In addition to a baseline scenario with a nursing home where patients can contact other patients, HCWs, and visitors, as described above (parameters as in Table 1), we study three different scenarios; (1) extreme scenarios of either a closed department, where patients cannot receive visitors and thus only contact other patients and HCWs, or an open department, where patients are assumed to have many contacts (also outside the nursing home) and, therefore, have the same probability of being infected as people in the community; (2) variations in HCW/patient ratios from 1 in the baseline scenario, consistent with our nursing home observations, to 1.5, and 0.67. In these simulations the number of HCWs is varied while the number of patients remains the same; (3) seasons with high and low influenza virus activity simulated with community epidemic curves with total attack rates of 5% and 15%, respectively, as compared to 10% in the baseline scenario.

In addition to the most plausible scenarios described here, we show simulations for some other scenarios in Text S1: a 60-bed nursing home department (Text S1 [Figure VI]); higher levels of immunity due to cross protection among HCWs (Text S1 [Figure VII]); a pandemic strain (Text S1 [Figure VIII]); an alternative mechanism of vaccine protection (Text S1 [Figure IX]); other infectiousness ratio between casual and close contacts (Text S1 [Figure X]); and a high vaccine efficacy for patients and HCWs (Text S1 [Figure XI]).

### Outcome

We study the relationship between the HCW vaccination rate and the fraction of patients that gets an influenza virus infection during the influenza season. The patient attack rate is defined as the total number of infections among patients divided by the total number of patients in the department during the study period. For every level of HCW vaccine uptake we perform 5,000 simulations (100 simulations × 50 parameter sets) of one nursing home department during one influenza season. We compute the arithmetic mean and median of infection attack rates among patients, the standard error of the mean, the range between 2.5-percentile and 97.5-percentile, and the proportion of infection attack rates of 0.3 or larger that we use as a proxy for the probability of a large outbreak. In addition to patient attack rates, we calculate the mean HCW attack rate, the mean number of introductions of influenza virus into the nursing home patient population (introduction rate), and the mean number of infections among patients following an introduction (patient attack rate per introduction). Finally, we calculate the absolute and relative risk differences of acquiring influenza virus infection for patients in departments where none or all of the HCWs are vaccinated.

### Herd Immunity

The concept of herd immunity has not been defined for a small population. In contrast to large populations, multiple introductions (with no or little transmission) in the nursing home can already affect a considerable fraction of the population, albeit still few patients. The distinction between these small outbreaks and larger outbreaks caused by substantial virus transmission is not always clear. In this study we use the absence (probability < 0.05) of large outbreaks (infection attack rate > 0.3) as a proxy for herd immunity.

### Power Analysis

To determine the number of departments needed for a trial to detect a difference between average HCW vaccination rates of 0 and 0.5, we use the power calculation for cluster randomized trials introduced by Kerry et al. [4,31] that is based on conventional power calculations [32]. We checked the accuracy of this equation using a simulation approach, see Text S1. When we use a significance level (α) of 5% and a power (1 − β) of 90% the number of departments required for each group is: *n* = 21(*s*_c_^2^ + *p*(1 − *p*)/*m*)/*d* ^2^, where *s*_c_^2^ is the between department variance and *p*(1 − *p*)/*m* the within department variance, *p* the fraction of individuals in the department with the outcome, *m* the number of individuals per department, and *d* the expected difference between the two groups.

## Results

### Baseline Scenario

**Figure 1:**
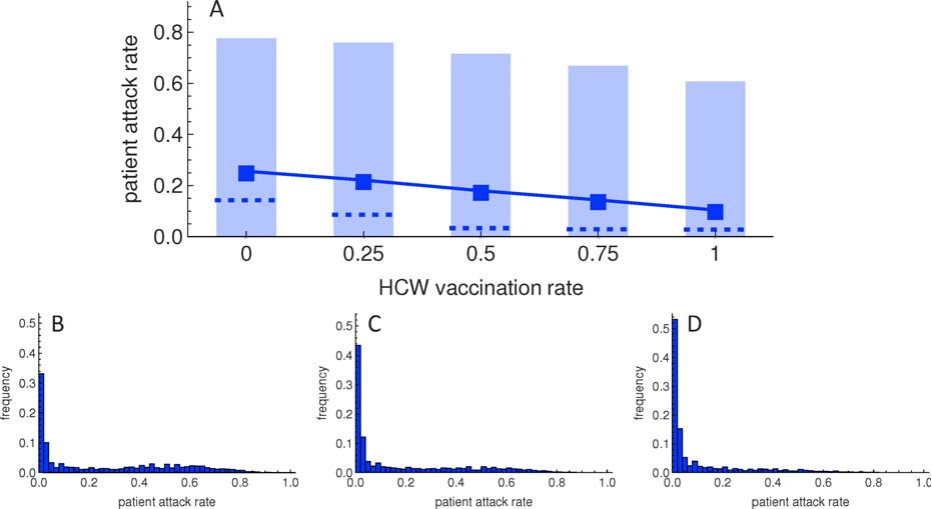
Influenza Virus Attack Rates among Patients for Increasing Health Care Worker Vaccination Rates (A) Increased vaccination of HCWs decreases the expected influenza virus attack rate among patients. Squares indicate the mean attack rates, dashed lines the median attack rates, and light blue boxes the 2.5th to 97.5th percentiles. (B–D) The distribution of the influenza virus attack rates among patients shifts to the left when vaccine uptake among HCWs is increased from 0, to 0.5, and 1 for (B), (C), and (D), respectively. Each distribution is based on 5,000 simulations.

An increase in HCW vaccine uptake decreases the expected influenza virus attack rate among nursing home patients (Figure 1). The relationship between the fraction of HCWs vaccinated and the mean patient attack rate appears linear. In the baseline scenario, with the parameter values as shown in Table 1, increasing HCW vaccination rate from 0 to 1 decreases the patient attack rate from 0.25 to 0.10, a risk difference of 0.15 (Figure 1A). Thus, approximately 60% of the patients that would have been infected without HCW vaccination are protected when all HCWs are vaccinated (relative risk 0.41), and seven HCWs have to be vaccinated to protect one patient from influenza virus infection. This NNT (number needed to treat) does not change with increasing vaccine uptake by HCWs, and no herd immunity is reached. The fraction of departments without infections among patients increases from 0.30 to 0.48, whereas the fraction of departments with a large epidemic (attack rates of more than 0.3) decreases from 0.41 to 0.14. Thus there is no evidence of herd immunity.

In the absence of HCW vaccination, the distribution of patient attack rates is bimodal, with peaks around attack rates of 0 and 0.6 (Figure 1B). With higher HCW vaccine coverage, mean and median patient attack rates decrease (Figure 1A), and the second peak disappears (Figure 1C and 1D). Due to stochastic variations the differences between patient attack rates are high and major outbreaks can occur at all levels of HCW vaccine uptake. The small standard error of the mean (< 0.0013 for all levels of HCW vaccine uptake) shows that we perform a sufficient number of simulations to obtain precise estimates of the mean, hence the observed variation is not due to sampling error. Additional simulations show that most of the variation is inherent to the chance events in the transmission process rather than parameter uncertainty (Text S1 [Figure II]; therefore these additional simulations justify the use of the mean and percentiles from the Latin hypercube parameter samples to illustrate the effect of HCW vaccine uptake on attack rate among patients in Figure 1). In a parameter uncertainty analysis the visitor frequency appears to have less impact on the attack rates among patients than the vaccine efficacies and the transmission probability (Text S1 [Figures IV and V]).

**Figure 2:**
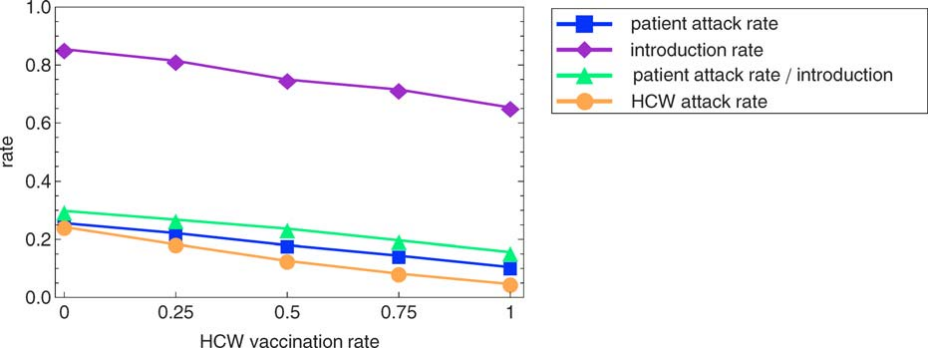
Effects of Increased Health Care Worker Vaccination on Influenza Virus Attack and Introduction Rates Increased vaccination of health care workers (HCWs) reduces the influenza virus attack rate among HCWs. It also reduces the rate of introduction of influenza virus into the patient population and the attack rate among patients following an introduction. As a consequence it reduces the total attack rate among patients. All relationships appear linear.

The effect of increased HCW vaccination on patient influenza virus attack rate can be attributed to a decrease in the number of introductions of influenza virus into the patient population as well as a decrease in the number of infections among patients following such an introduction (Figure 2). Both reductions are caused by a decreased number of influenza virus infections among HCWs (Figure 2).

### Scenario Analyses

**Figure 3:**
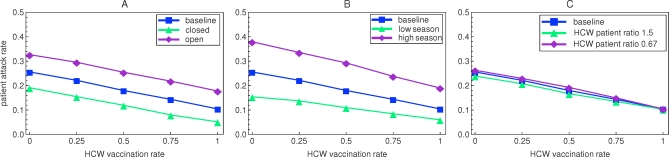
Effects of Increased Health Care Worker Vaccination on Patient Attack Rates in Different Scenarios For all scenarios under study, the influenza virus attack rate among patient decreases in an approximately linear way when the health care worker vaccination rate is increased. (A) The expected attack rates for the open and closed departments, where patients have many or no contacts with individuals from the community, respectively. (B) The attack rates for seasons with high (15% community attack rate) and low (5% community attack rate) influenza virus activity. (C) The attack rates for departments with high and low HCW/patient ratios.

In the scenarios with an open and closed department the absolute change in the expected patient attack rate is similar to that in the baseline scenario (Figure 3A). The relative change caused by an increase in HCW vaccination rate from 0 to 1 is highest for the closed department (73%). Also in scenarios with higher and lower influenza virus activity, the decrease in patient attack rate upon increased vaccination of HCW is approximately linear (Figure 3B). The absolute decrease in patient attack rate is highest in the season with high influenza activity (0.19), but the relative decrease is lower than that in the baseline scenario (50%). In scenarios with other HCW/patient ratios (1.5 and 0.67, respectively), the fraction of HCWs that has to be vaccinated to protect one patient is similar to what we observed in the baseline scenario (Figure 3C). The absolute number of HCWs to be vaccinated is however different (11 and five, respectively). Simulations for a 60-bed department, alternative vaccine efficacy mechanism, and some more parameter variations also give qualitatively similar results (Text S1 [Figure VI]). In case of pandemic influenza, with full absence of immunity in the population, we analyze a best case scenario in which we assume that a vaccine is available with equal efficacy as the vaccines for seasonal strains. Without vaccination of HCWs, in this scenario, major outbreaks occur in all departments with an average patient attack rate of 0.59. When the HCW vaccination rate is increased to 1, the patient attack rate decreases to 0.37, but large outbreaks still prevail (Text S1 [Figure VIII]).

### Power Analyses

A power calculation for cluster randomized trials using the effect estimates and variances of the simulations with the default parameter set (see Text S1) reveals the need of 184 departments per arm to allow detection of a statistically significant difference (α = 5%) in patient attack rates between departments with HCW vaccination rates of 0 and 0.5 with a 90% power (Text S1 [Figure III]). With a simulation approach we find a need of 169 departments to detect such a difference at the 5% level with 90% power. In both power calculations we do not take into account variation due to differences between departments (e.g., size, HCW/patient ratios, health state of patients), influenza seasons, and vaccine matching, which would further increase the number of departments required.

## Discussion

Our model reveals a linear relationship between the number of HCWs vaccinated and the expected number of influenza virus infections among patients in a nursing home department. No threshold for herd immunity can be detected and even when HCW vaccine uptake is maximal, due to stochastic effects that are inherent to the transmission process, the variation in patient attack rates is high and large outbreaks can occur. In fact, the value of small-sized clinical trials in estimating the protective effect of HCW vaccination on patient outcome seems questionable due to the large impact of chance effects within the small environment of nursing home departments.

In order to appreciate the results of our modeling study some possible limitations need to be addressed. First, we assume that all individuals in the model, whether patient, HCW, or visitor are equally infectious or susceptible. In addition, all individuals from the same group are assumed to have similar contact probabilities. Both assumptions decrease the heterogeneity in the system, which might have slightly increased the probability of a major outbreak [12]. Second, we model the nursing home department as an independent institute and neglect its potential connections with other departments. In fact, within a nursing home some of the introductions of influenza virus in a department may come from another department. Vaccination of HCWs in all departments reduces the transmission risk in each of them, and, thus, provides indirect protection. Therefore, the protective effect of HCW vaccination per given vaccine will be higher on the level of an entire nursing home, which might have led to an underestimation of the impact of HCW influenza vaccination. Third, we only consider departments of 30 beds. However, simulations of larger nursing home departments (see Text S1 [Figure VI]) reveal that department size has little impact on the qualitative results. Fourth, the simulated infection attack rates only give an approximate indication of the corresponding influenza-like illness (ILI) attack rates. To convert infection attack rates to ILI attack rates, we use two empirical findings: influenza virus infection leads to illness in approximately 50% of the cases [33]; approximately 50% of observed ILIs is caused by influenza virus infection [34,35]. Taken together, the influenza virus attack rate roughly approximates the observed ILI attack rate.

To our knowledge our model is the first to explore the effect of HCW vaccination on the occurrence of influenza virus infections in nursing home patients. The major advantage of our model over previously performed experimental and observational studies [3–5,36,37] is the possibility to simulate various levels of HCW vaccine uptake and perform multiple simulations to minimize the influence of chance effects. Therefore, it can be used to reinterpret the outcome of small-scale clinical trials. We find very wide distributions of patient attack rates that agree with reported differences in outbreak sizes and attack rates between departments and seasons [29]. Also due to the diversity in outcomes, all available data on attack rates in nursing homes are in line with our simulation results. Our model suggests that for plausible regions in the parameter space there is no herd immunity threshold above which all patients are protected as was hypothesized before [10,38]. To the contrary, every additional HCW vaccination protects an additional fraction of patients and therefore increasing HCW vaccination rate from 0.8 to 0.9 is as important as increasing it from 0.1 to 0.2. Consequently, if HCW vaccination is adopted as a policy, benefits are to be expected for every additional vaccination as long as 100% coverage is not achieved. The unexpected absence of herd immunity for nursing home populations can be explained by two facts. First, due to the low efficacy of the influenza vaccine, especially among the elderly, the fraction of susceptible individuals remains substantial even with high vaccination rates. Second, the nursing home department is not a large closed population, for which the concept of herd immunity has been established. Instead it is a small population with many links, through HCWs and visitors, to a larger community where an influenza epidemic is ongoing.

Our study further demonstrates the large impact of stochastic events on patient attack rates, which is of immediate concern for both the interpretation of previously performed HCW vaccination trials and the design of future experimental studies. A power calculation for cluster randomized trials (α = 0.05, β = 0.10) [31] in which we use the variances and effect estimates obtained from 5,000 simulations with one parameter set, reveals the need of 184 departments per arm to detect a significant difference in patient attack rates between departments with HCW vaccination rates of 0 and 0.5. This suggests that the previously performed trials on HCW vaccination, with six to 23 departments per arm [3–5], were underpowered and cannot be assumed to give a precise effect estimate of HCW vaccination. The difference between the small confidence intervals around the effect estimates in these studies and the large variance predicted by our model, might be explained by a difference between the measured sample variance and the true population variance. With small sample sizes, the sample variance can deviate substantially from the population variance [39]. Moreover, the performed trials were based on secondary endpoints such as ILI, influenza-related hospital admissions, or (all cause) mortality rather than influenza infection, which further reduces their power, since these endpoints occur less often than influenza infection and the difference between control and intervention groups is expected to be smaller. These findings should be taken into account when designing future studies to demonstrate the generally presumed benefit of HCW vaccination.

## Supporting Information

Text S1 Detailed Description of Mathematical Model (1.2 MB DOC)

## Abbreviations

HCW: health care worker
ILI: influenza-like illness
