# Supplementary material for: The Effects of Influenza Vaccination of Health Care Workers in Nursing Homes: Insights from a Mathematical Model
Source: PLoS Med. 2008 Oct 28;5(10):e200. doi: 10.1371/journal.pmed.0050200 (PMC2573905; doi:10.1371/journal.pmed.0050200)
Supplement: Text S1 — (1.2 MB DOC) [file pmed.0050200.sd001.doc]

**Supporting Information – Text S1**

**The Effects of Influenza Vaccination of Health Care Workers in Nursing Homes: Insights from a Mathematical Model**

Carline van den Dool1*, Marc J. M. Bonten1,2, Eelko Hak1, Janneke C.M. Heijne3, Jacco Wallinga1,3

1 Julius Center for Health Sciences and Primary Care, University Medical Center Utrecht, Utrecht, The Netherlands; 2 Department of Medical Microbiology, University Medical Center Utrecht, Utrecht, The Netherlands; 3 Center for Infectious Diseases Epidemiology, National Institute for Public Health and the Environment, Bilthoven, The Netherlands;

*Corresponding author. c.vandendool@umcutrecht.nl.

**Table of Contents**

1. Model and simulation algorithms

2. Observed contact patterns in a nursing home department

3. Simulations for a single parameter set

4. Power calculations for a single parameter set

5. Multivariate uncertainty analyses

6. Additional scenarios

**1. Model and simulation algorithms**

**Influenza in the community**

The spread of influenza in the community of 100,000 individuals outside the nursing home is described by four variables: s, the proportion of susceptible individuals in the community, e, the proportion of exposed individuals in the community, i, the proportion of infectious individuals in the community, r, the proportion of recovered and immune individuals in the community. We determine the time course of these variables by simulating an influenza epidemic with a deterministic so-called “susceptible-exposed-infectious-recovered” (SEIR) model, as given by the system of ordinary differential equations below:

ds/dt = – λ s

de/dt = λ s – σ e

di/dt = σ e – γ i

dr/dt = γ i

force of infection λ = β i .

The values of the parameters σ and γ are chosen such that the average generation time equals 2.8 days, the value that was observed in a household study [1,2]. The value of the contact parameter β varies over time to mimic the effect of seasonality. The resulting basic reproductive number (R0), has an average value of 1.5. We start simulating the epidemic at t= –55 (“autumn”) with one infectious individual, and assume that seventy percent of the population is susceptible and thirty percent of the population is immune as has been seen in previous studies [3,4]. Furthermore, these numbers are consistent with the assumptions that immunity lasts approximately 4 years, and the average annual infection attack rate is around 10%. We use t = 0 as the start of the influenza season and t = 80 as the end of the influenza season. The simulated daily influenza infection incidence, λ s, and prevalence i, are shown in Figure S1.

**Figure I**. Incidence and prevalence of influenza virus infections in the community.

**Influenza in the nursing home**

The nursing home has m health care workers (HCWs) and n beds. Each HCW is labeled j=1,2,…m. The work schedule of the HCWs is indicated by a function w(t,j) such that w(t,j)=1 if the jth HCW is at work at time t and w(t,j) = 0 if the ith HCW is not at work at time t. This work schedule is designed such that at each day 5 HCWs work during the day shift, 3 during the evening shift and 1 during the night shift. All HCWs are dedicated to specific shifts according to a weekly schedule that is drawn randomly before every simulation. No distinction is made between week days and weekend. There are no changes in the HCW pool and the working schedule during the simulated period.

**A stochastic simulation model of influenza epidemics in a nursing home: state variables**

The state of each bed (j =1,2…n) is indicated as x(t,j) and consists of one variable that takes one out of five possible values: {vacant, susceptible, exposed, infectious, recovered}. The state of each HCW (j = 1,2,…m) is indicated as y(t,j) and consists of two variables. The first variable takes one out of two possible values: {at work, not at work}. The second variable takes one out of four possible values: {susceptible, exposed, infectious, recovered}.

At each moment t, the state of the system is completely characterized by the state vectors x, y. For convenience, we also use aggregate variables whose values are completely determined by the state variables: the number of patients that are infectious at time t, I­­P(t); the number of HCWs at work that are infectious at time t, IH(t)

**Algorithm**

A stochastic model specifies the state variables in the model, the rules for changing the value of these state variables, and the rates at which the state variables change over time. Ideally, such a model is solved using an exact algorithm that keeps track of each discrete event at which a state variable changes its value. The computational requirements of such an exact algorithm can become prohibitively large if we want to explore the model outcome for a large number of evaluations and for a wide range of parameter sets. Therefore, we use a discrete-time simulation approach that is more computationally efficient. For each time step Δt we keep track of the number of discrete events that happened over this time step, and update the state variables accordingly. The error made in such a discrete-time simulation approach is kept small by choosing small time steps. In our case, we set the time step to 8 hours (equal to a working shift). We calibrate one parameter (the probability of transmission per contact) to obtain a preset average model outcome (the infection attack rate without interventions). This calibration further reduces the impact of possible errors. Although the discrete time approach does introduce a small inexactness in the simulated outcome, its computational efficiency allows us to thoroughly explore the plausible regions of the parameter space.

**Update rules**

At each time step Δt the values of the state variables are updated to account for transitions. The probability of each of these transitions to occur is specified according to the rules in Table I.

**Initial conditions**

At the start of the influenza season each HCW has a probability rc of being immune due to cross protection. With probability u1 each patient has been vaccinated at the start of the influenza season (t=0) with vaccine efficacy ve1. With probability u2 each of the HCWs is vaccinated at the start of the influenza season (t=0) with vaccine efficacy ve­2.

**Table I.** Transitions and probabilities in the model

|  | |  | | | | Transition | | Probability# |
| --- | --- | --- | --- | --- | --- | --- | --- | --- |
| Patient flow | | | | | |  | |  |
|  | | discharge or death | | | | P(x(t+Δt,j) = vacant | x(t,j) = ¬vacant)¶ | | μ Δt |
|  | | admission | | | | P(x(t+Δt,j) = S | x(t,j) = vacant) | | (1 - u1 ve1) n μ Δt |
|  | | admission | | | | P(x(t+Δt,j) = E | x(t,j) = vacant) | | e n μ Δt |
|  | | admission | | | | P(x(t+Δt,j) = I | x(t,j) = vacant) | | i n μ Δt |
|  | | admission | | | | P(x(t+Δt,j) = R | x(t,j) = vacant) | | u1 ve1 n μ Δt |
| HCW flow | | | | | |  | |  |
|  | | working | | | | P(y(t+Δt,j) = {at work,·})‡ | | w(t+Δt,j) |
|  | | at home | | | | P(y(t+Δt,j) = {not at work,·}) | | 1-w(t+Δt,j) |
| Course of infection of patients | | | | | |  | |  |
|  | | infection | | | | P(x(t+Δt,j )= E | x(t,j) = S) | | λ1(t) Δt |
|  | | becoming infectious | | | | P(x(t+Δt,j) = I | x(t,j) = E) | | σ Δt |
|  | | recovery | | | | P(x(t+Δt,j) = R | x(t,j) = I) | | γ Δt |
| Course of infection of HCWs | | | | | |  | |  |
|  | | infection at work | | | | P(y(t+Δt,j) = {·,E} | y(t,j) = {at work, S}) | | λ2(t) Δt |
|  | | infection at home | | | | P(y(t+Δt,j) = {·,E} | y(t,j) = {not at work, S}) | | λ s Δt |
|  | | becoming infectious | | | | P(y(t+Δ t,j) = {·,I} | y(t,j) = {·, E}) | | σ Δt |
|  | | recovery | | | | P(y(t+Δ t,j) = {·,R} | y(t,j) = {·,I}) | | γ Δt |
|  | |  | | | |  | |  |
| Forces of infection | | | | | |  |  |  |
|  | For patients | | | | |  |  | |
|  | | | Day | | λ1(t) = λ11+λ12+λvis | | λ11= (π11 ρ +(1-π11)) pc c11 IP | |
|  | | | Evening | | λ1(t) = λ11+λ12+λvis | | λ12= (π12 ρ +(1-π12)) pc c12 IH | |
|  | | | Night | | λ1(t) =Ind(x(t,j+Neighbor(j))=I) pc+λ12 * | | λvis=g ρ pc i | |
|  | For HCWs | | | | |  |  | |
|  | At work | | | λ2(t )= λ21 +λ22 | | | λ21= (π21 ρ +(1-π21)) pc c21 IP | |
|  |  | | |  | | | λ22= (π22 ρ +(1-π22)) pc c22 IH | |

# see Table II for the meaning on the symbols used

¶ we use ¬vacant to denote any possible state except vacant

‡ we use {at work, ·} to denote any possible state where first state variable is equal to the state at work

* we use Ind(x(t,j)=J) to mean an indicator function that returns the value 1 if its argument is a correct expression, and 0 if its argument is false; we use Neighbor(j)=j-1+2 Mod[j,2] as a function that returns the index of the room mate of the patient, such that Neighbor(1)=2, Neigbor(2)=1

**Table II.** Parameters in the model

| Symbol | Parameter | Default | Units | Ref |
| --- | --- | --- | --- | --- |
| n | Number of beds | 30 |  |  |
| m | Number of HCWs | 30 |  |  |
| Δt | Time step (shift) | 8 | hours |  |
| T | Minimum duration of simulation | 80 | days |  |
| μ | Discharge/mortality rate | 1/425 | day-1 | [5,6] |
| σ | Rate of becoming infectious after infection | 1/1.4 | day-1 | [1,2] |
| γ | Infection recovery rate | 1/1.4 | day-1 | [1,2] |
| rc | Fraction of HCW immune due to through cross- protection | 0.3 |  | [3,4] |
|  | Vaccination uptake patients | 0.75 |  | [7] |
|  | Probability of contact between |  |  |  |
| c11 | Patient – patient | 0.07 | shift-1 |  |
| c12 | HCW – patient | 0.52 | shift-1 |  |
| c22 | HCW – HCW | 0.91 | shift-1 |  |
|  | Probability of close contact between |  |  |  |
| π11 | Patient – patient | 0.06 | contact-1 |  |
| π12 | HCW – patient | 0.69 | contact-1 |  |
| π22 | HCW – HCW | 0.32 | contact-1 |  |
| ρ | Close/casual transmission probability ratio | 2 |  |  |
|  | Vaccine efficacy (against infection) |  |  |  |
| ve1 | Patients | 0.25 |  | [8] |
| ve2 | HCWs | 0.73 |  | [9] |
| pc | Transmission probability casual contact | 0.13 | contact-1 |  |
| g | Average number of visitors | 0.7 | ­patient-1 day-1 | [10] |
| u2 | Vaccine uptake HCW | 0 – 1.0 |  |  |

**Duration of simulations**

We use a time step Δt of 8 hours, which is equal to a HCW’s working shift. In all runs, the simulations are continued until the simulated period equals at least 80 days (the length of the influenza season). If there are still infected individuals around after 80 days, simulation is continued until the epidemic is extinct.

**2. Observed contact patterns in a nursing home department**

In two nursing home departments we interviewed HCWs and patients and asked for the numbers of different contacted individuals per day, categorized by the type of contacted

**Table III.** Observed number of contacts

| Symbol | | Per Patient | Per HCW |
| --- | --- | --- | --- |
| Non-physical contacts | |  |  |
|  | Number of patients | 3.9 | 16.2 |
|  | Number of HCWs | 4.5 | 7.3 |
| Physical contacts | |  |  |
|  | Number of patients | 0.2 | 9.9 |
|  | Number of HCWs | 3.4 | 2.3 |

**Table IV.** Observed contact rates

| Symbol | patient | HCW |
| --- | --- | --- |
| patient | 0.13 | 0.54 |
| HCW | 0.50 | 0.91 |

**Table V.** Probability that a contact is close (physical) rather than casual (non-physical)

| Symbol | patient | HCW |
| --- | --- | --- |
| patient | 0.06 | 0.61 |
| HCW | 0.76 | 0.32 |

individual (HCW or patient) and type of contact (physical, such as touching; non-physical, such as having a conversation). The tentative results of this small survey are presented in table III. We derive the contact rates by dividing these reported numbers by the numbers of HCWs and patients present in the nursing homes (Table IV). As a check of the accuracy of reporting in this survey, we compare the contact rate between HCWs and patients as reported by HCWs (0.54) with the same contact rate reported by patients (0.50). Because the contacts are reciprocal, the contact rates should be similar. We see this is almost the case, which indicates that reporting has been accurate. We take the average of both rates as the best estimate of the contact rate for our simulation study. We derive the probability that a contact is physical (a close contact) rather than non-physical (casual) by taking the ratio of reported physical contacts to all reported contacts (Table V). As a second check of the accuracy of reporting in this survey, we compared the probability of close contact as reported by HCWs (0.61) with the same probability as reported by patients (0.76). Both measures are approximately similar, which indicates accurate reporting in this survey, but a higher accuracy for contact rates than for probability of close contacts. We take the average of both probabilities as the best estimate of the probability of close contact for our simulation study.

**3. Simulations for a single parameter set**

We parameterize the stochastic model with the default parameter values in table II, and perform 5000 simulations for each value of the vaccination rate of HCWs (Figure II). These simulations show the range in outcomes that is due to chance events in the transmission process. As compared with the simulations for 50 different parameter sets in the main text, figure 1, we see that the range from 2.5 to 97.5 percentile for a single parameter set is only slightly smaller than for 50 parameter sets. Thus most of the variation in attack rates appears to be due to stochastic effects and not to the variation in parameter values.

**Figure II**. Influenza virus attack rates among patients for increasing health care worker vaccination rates for the best estimate parameter set.

**4. Power Calculations for a single parameter set**

To check the accuracy of the power calculation for cluster randomized trials, when applied to study infectious diseases in small populations, we perform an alternative Monte Carlo power calculation in which we simulate randomized trials by taking samples of different size from a large dataset of 5000 outcomes for departments with a HCW vaccination rate 0 and a HCW vaccination rate 0.5. For sample sizes increasing from 10 to 220 we observe in which fraction of the simulated trials we can detect, using a t-test, a significant difference at 5 % significance level between the mean attack rates in the groups with HCW vaccination rate 0 and 0.5. Figure III shows the relationship between sample size and power according to our simulation study. A sample size of 169 departments is required to detect the difference with a 90% power. This relationship is accurately approximated by the calculation for cluster randomized trials, where we find that 184 departments per group are required to detect the difference between the two groups with a 90% power.

**Figure III.** Power calculation based on simulations (dotted blue line) and using the standard equation for cluster randomized trials (solid purple line).

**5. Multivariate uncertainty analyses**

We use Latin hypercube sampling to do an uncertainty analysis for four parameters. The results are shown in figure IV. The HCW vaccination rate is kept constant at 0 (left column), 0.5 (middle column) and 1 (right column). We vary the patient vaccine efficacy over a range form 0 to 0.5 (top row), the HCW vaccine efficacy over a range form 0 to 0.9 (second row), the transmission probability from 0.10 to 0.16 (third row) and visitor frequency from 0.4 to 1.0 (bottom row). The results show that the influenza virus attack rate among patients is highest when vaccine efficacy for patients is low, especially in the departments where HCW vaccine uptake is low. At higher levels of HCW vaccine uptake we see a negative correlation between the HCW vaccine efficacy and patient attack rate. As expected there is a positive relation between the transmission probability and the influenza virus attack rate among patients. The number of visitors does not appear to have a large impact on the influenza virus attack rate among patients.

**Figure IV.** Results of multivariate uncertainty analyses. We vary the parameters health care worker (HCW) vaccination rate (columns) simultaneous with patient vaccine efficacy (top row), HCW vaccine efficacy (second row), transmission probability (third row) and visitor frequency (bottom row).

We also study the correlations between the parameter values and the maximal change in influenza virus attack rate among patients, resulting from an increase in HCW vaccination rate from 0 to 1 (Figure V). The effect of HCW vaccination is negatively associated with the vaccine efficacy for patients. It is positively associated with the vaccine efficacy for HCWs and, though somewhat less strongly, with the transmission probability. For the studied parameter values, there is no clear association between the change in patient influenza virus attack rate and the visitor frequency.

**Figure V.** Uncertainty analyses for the maximal effect of health care worker (HCW) vaccination. We vary the parameters patient vaccine efficacy, HCW vaccine efficacy, transmission probability and visitor frequency and compare the change in patient attack due to an increase in HCW vaccination rate from 0 to 1.

**6. Additional scenarios**

Here we present the results of some additional scenario analyses to show the robustness of our model.

**60-bed department**

To determine the effects of unit size we repeat the simulations for a 60-bed department. In this scenario 10 HCWs work during the day shift, 6 during the evening and 2 during the night. We use the same contact structure for patients and HCWs as before, yielding the same number of contacts per shift. Yet, because of the higher number of individuals in the department, the probabilities to contact a specific other patient or HCW are of course lower. In this scenario the average influenza virus attack rates are higher than in the 30-bed nursing home department (Figure VI). Raising HCW vaccination rate from 0 to 1 linearly decreases the influenza virus attack rate among patients from 0.36 to 0.12 (RR 0.33, NNT 5 (4.23)). The relative risk reduction for patients due to increased HCW vaccination is thus similar for the two department sizes but due to the higher attack rates in the bigger department the absolute decrease in attack rate is larger.

**Figure VI.** Influenza virus attack rates among patients for increasing health care worker vaccination rates for a 60-bed department

**High levels of adult immunity due to cross-protection from earlier infection**

To determine the impact of our assumption that 30% of the healthy adults is immune due to cross protection, we here show the results of scenarios in which the immunity (before vaccination) is 0.5 and 0.7, respectively (Figure VII). In these scenarios, the influenza virus attack rate among patients in the absence of HCW vaccination is lower than in the baseline schenario due to the higher number of immune HCWs. Increasing vaccination of the HCWs further decreases the patient attack rate. In both scenarios, for all levels of HCW vaccination, the fraction of large outbreaks is more than 0.05 and thus, no herd immunity is achieved.

**Figure VII.** Influenza virus attack rates among patients for increasing health care worker vaccination for different initial levels of immunity among HCWs before vaccination (due to cross-protection from previous infections).

**Pandemic**

In light of the present pandemic threat, we study the potential protective effect for nursing home patients of vaccinating HCWs during a pandemic. Because we do not know the efficacy of a future pandemic vaccine, we study the best case scenario in which the vaccine

efficacy of the pandemic vaccine is equal to that of the yearly influenza vaccines. We assume the only difference between the pandemic and the epidemic strains is the absence of prior immunity in the population to the pandemic one. Our simulations show that in this scenario major epidemics occur in all nursing home departments (Figure VIII). Although HCW vaccination can reduce the attack rate among patients to some extent (from 0.59 to 0.37), major outbreaks cannot be prevented due to the high prevalence of influenza in the community (total attack rate 0.55) and the associated high introduction rate into the nursing home.

**Figure VIII.** Pandemic influenza virus attack rates among patients for increasing health care worker vaccination rates.

**Vaccine efficacy – alternative mechanism of action**

Unfortunately, not so much information is available concerning the mechanism of protection of vaccines. We use an all or nothing model, since we assume that an individual who does not get infected the first time after contact with the influenza virus is even less likely to become infected the second time, when all defense mechanisms have already been triggered. To evaluate the impact of this choice we perform simulations with an alternative model in which vaccination reduces the transmission probability of the virus during an infectious contact. In other words, a vaccinated individual can still become infected but the average number of infectious contacts necessary for infection will be higher than for a non-vaccinated individual. We assume the protection per contact to be 30% and 80% for patients and HCWs respectively, which leads to vaccine efficacies over the season of 11% and 89%. Because of the lower overall patient vaccine efficacy the initial patient attack rates are higher than in the baseline scenario (Figure IX), but again increased HCW vaccination causes a similar linear decrease in the patient attack rate.

**Figure IX.** Patient influenza virus attack rates for increasing health care worker vaccination compared for the baseline scenario and an alternative mechanism of vaccine efficacy

**Infectiousness ratio casual and close contacts**

We assume a close contact is twice as infectious as a casual contact. To determine the impact of this choice on the model outcome we perform simulations in which the close contact is 1.5 and 2.5 times as infectious as the casual contact. Since we do not recalibrate

the transmission probability the attack rates for this scenario are somewhat lower and higher, respectively, than for the baseline scenario (Figure X). However, the effect of HCW vaccination is comparable.

**Figure X.** Patient influenza virus attack rates for increasing health care worker vaccination compared for different infectiousness ratios for casual and close contacts (ρ).

**High vaccine efficacy for patients and HCWs**

In a scenario where the vaccine efficacy for patients is 70% and for HCWs 90 %, we again find a linear relation between the fraction of HCWs vaccinated and the average patient attack rate (Figure XI). In this scenario the probability of a major outbreak (>0.3) can be reduced to 2.6%. Thus in a scenario wilth high vaccine efficacies herd immunity (<5% major outbreaks) can be achieved.

**Figure XI.** Patient influenza virus attack rates for increasing health care worker vaccination in a scenario with high vaccine efficacy.

Reference List

1. Hirotsu N, Ikematsu H, Iwaki N, Kawai N, Shigematsu T et al. (2004) Effects of antiviral drugs on viral detection in influenza patients and on the sequential infection to their family members--serial examination by rapid diagnosis (Capilia) and virus culture. International Congress Series 1263: 105-108.

2. Wallinga J, Lipsitch M (2007) How generation intervals shape the relationship between growth rates and reproductive numbers. Proc Biol Sci 274: 599-604.

3. Rvachev A, Longini IM (1985) A mathematical model for the global spread of influenza. Mathematical Biosciences 75: 3-22.

4. Cauchemez S, Valleron AJ, Boelle PY, Flahault A, Ferguson NM (2008) Estimating the impact of school closure on influenza transmission from Sentinel data. Nature 452: 750-754.

5. CTG ZAio (2006) Doelmatigheid verpleeghuizen in relatie tot verantwoorde zorg onderzocht.

6. JCM Tekstproducties (2006) Arcares jaarverslag 2005.

7. Hayward AC, Harling R, Wetten S, Johnson AM, Munro S et al. (2006) Effectiveness of an influenza vaccine programme for care home staff to prevent death, morbidity, and health service use among residents: cluster randomised controlled trial. BMJ 333: 1241.

8. Jefferson T, Rivetti D, Rivetti A, Rudin M, Di Pietrantonj C et al. (2005) Efficacy and effectiveness of influenza vaccines in elderly people: a systematic review. Lancet 366: 1165-1174.

9. Jefferson T, Rivetti D, Di Pietrantonj C, Rivetti A, Demicheli V (2007) Vaccines for preventing influenza in healthy adults. Cochrane Database Syst Rev CD001269.

10. de Klerk M (2005) Ouderen in Instellingen. 26-29.
